# Tuberculosis among Jimma University Undergraduate Students: First Insight about the Burden of Tuberculosis in Ethiopia Universities—Cross-Sectional Study

**DOI:** 10.1155/2017/9840670

**Published:** 2017-10-24

**Authors:** Deneke Wolde, Mulualem Tadesse, Kedir Abdella, Gemeda Abebe, Solomon Ali

**Affiliations:** ^1^Department of Medical Laboratory Technology, Hossana College of Health Sciences, Hossana, Ethiopia; ^2^Department of Medical Laboratory Science, Institute of Health, Jimma University, Jimma, Ethiopia; ^3^Mycobacteriology Research Center, Jimma University, Jimma, Ethiopia; ^4^AERAS Africa, Rockville, MD, USA

## Abstract

**Background:**

Ethiopian universities are facilities where students live in relative overcrowding condition. This might favor the chance of tuberculosis transmission among students. This study was done to determine the magnitude and associated factors of tuberculosis among Jimma University students.

**Methods:**

A cross-sectional study was done from February 2015 to July 2015. Hundred twenty-nine consented participants were interviewed using structured questionnaire. Biological specimens were collected and cultured on Mycobacterium Growth Indicator Tube.* Mycobacterium tuberculosis* complex verification was done by SD BIOLINE TB Ag MPT64 Rapid test. Frequency distribution, logistic regression, and independent sample *t*-test were used to analyze the data using SPSS Version 20.

**Result:**

Magnitude of all forms of tuberculosis among Jimma University undergraduate students was 209.1 per 100000-student population. Contact history [AOR: 4.76, 95% CI (1.31–17.31)], smoking [AOR: 6.67, 95% CI (1.51–29.44)], khat chewing [AOR: 5.56, 95% CI (1.66–18.69)], and low body mass index [AOR: 5.37, 95% CI (1.46–19.78)] were determinants of tuberculosis.

**Conclusion:**

The magnitude of tuberculosis among Jimma University undergraduate students is high. TB is associated with previous tuberculosis patient contact and behavioral factors. Hence, students with these risk factors should be given enough attention for the control of TB in Jimma University.

## 1. Introduction

In Ethiopia, TB is the leading cause of morbidity, the third cause of hospital admission, and the second cause of death [1]. Ethiopia is ranked as fourth from African and tenth from the world based on the number of TB cases occurring annually [2]. Ethiopian national prevalence survey conducted in 2010/2011 estimated the prevalence of bacteriologically confirmed TB as 277 per 100,000 [3]. However prisons, universities, refugees, and military camps were not addressed in this survey. These groups of population could be considered as high risks especially considering the expected overcrowding, stress, and nutritional conditions.

It is common in Ethiopian universities to see large number of undergraduate students sharing the same dormitory, learning at the same lecture hall, and sharing the same library halls for reading. Currently in Jimma University, an average of six to eight students are sharing one dormitory room, 60 to 100 students are attending lectures in one classroom, and 500 to 700 students dine in the same hall. Considering these facts one can assume that the risk of TB transmission among Ethiopian University students might be high. This risk can also be further fueled by the existing stress condition which is mainly associated with the academic burden.

The risk of tuberculosis has also been associated with smoking, chronic lung diseases, alcoholism, malnutrition [4–6], poor ventilation, overcrowding, and psychosocial circumstances [5]. Prolonged contacts to patients with TB in a variety of settings are also described as a substantial risk for tuberculosis through increasing the probability of bacteria spread [7, 8].

Tuberculosis in the educational setups is not a new idea. Previous studies done elsewhere documented an outbreak of latent TB infection in colleges and boarding schools [9–13]. In Ethiopia, also previous retrospective study done at Gondar University, Addis Ababa University, and Addis Ababa Science and Technology University reported prevalence of 354.66, 521.6, and 1098.1 per 100000-student population, respectively [14, 15]. However, these studies have calculated the prevalence from registration book and the retrospective study design used in these studies could not allow investigating other associated factors of TB.

To the best of our knowledge, no well-designed previous study has been done to properly describe the magnitude of TB and associated factors in Ethiopia universities. Therefore, this study was done to give the first insight about TB magnitude and associated factors among Jimma University undergraduate students.

## 2. Methods

### 2.1. Setting and Study Population

Jimma University is one of the biggest academic institutes in Ethiopia. It is located in the city of Jimma, 355 kilometers away from the capital Addis Ababa in southwest direction. The university is organized in three campuses: Main Campus, Agriculture and Veterinary Medicine Campus, and Jimma Institute of Technology Campus which are situated at different locations of Jimma city. The university currently has 10 Ph.D. programs, 102 masters programs, 52 undergraduate programs, and 8 pedagogical certification programs [10]. The total number of under graduate students in the university during the 2014/2015 academic year was 19,598.

### 2.2. Study Design and Inclusion Criteria

Cross-sectional study was done from February 1 to June 30, 2015, on all undergraduate students with cough of ≥2 weeks and/localized pain/swelling on different sites with general sign and symptom of TB. Students who were on treatment at the beginning of the study, extension students, and those students who were unwilling to participate on the study were excluded.

### 2.3. Data Collection

All students who had visited any of the three (Main, Agriculture and Veterinary Medicine, and Jimma Institute of Technology) student clinics with sign of tuberculosis were sent to Jimma University Hospital Medical Outpatient Department (OPD) for further evaluation. Students who fulfill any of the inclusion criteria were requested for their consent to take part in this study after giving them a detailed explanation about the purpose, benefit, and risk of participation in this study. Sociodemographic and some TB associated factors and clinical data were collected from consented participants by questioner. Then, participants were sent to Jimma University Mycobacteriology Research Centre (MRC) with request form for sputum microbiological examination.

### 2.4. Laboratory Methods

#### 2.4.1. Specimen Collection and Transportation

Participants were provided three falcon tubes to collect three consecutive (spot-morning-spot) sputum specimens [1]. Proper sputum collection technique was demonstrated by study laboratory staff to collect quality sputum. In addition, specimens from different body sites were collected by pathologist and sent to MRC aseptically. All specimens received in the laboratory were checked for quality and registered on laboratory log book.

#### 2.4.2. Specimen Processing and Culturing

The quality of sputum was checked upon reception, saliva and soil contaminated specimens were rejected, and participants were asked to bring another specimen. Ziehl-Neelsen (ZN) staining microscopy was done on one early morning and spot specimen for immediate delivery of result. Fine needle aspirate (FNA) samples were also collected from a swollen superficial lymph node of EPTB suspected patients by pathologists. The first few drops of aspirate were placed on a clean slide for FNA cytology and the remaining were sent for culture.

Morning sputum and all other body fluids were processed by NaOH-NALC method for culture. Five hundred microliter processed specimens were inoculated on Mycobacterium Growth Indicator Tube (MGIT) and incubated in the BACTEC MGIT^960^ (Becton Dickinson, USA) system as per manufacturer recommendation [16]. By the time the machine flags positive, the tubes were withdrawn from the BACTEC MGIT^960^ system and then were inoculated on blood agar and examined by ZN Microscopy to differentiate contamination from real growth. SD BIOLINE TB Ag MPT64 Rapid test was done for tubes which were positive for acid fast bacilli under microscopy and no growth on blood agar after 48 hrs incubation to verify MTBC. Contaminated samples were reprocessed again and inoculated on liquid media and placed in the MGIT^960^ system.

### 2.5. Data Analysis and Interpretation

After collection, the data were checked for completeness and they were categorized, coded and entered, and analyzed using Statistical Package for Social Sciences (SPSS) version 20. Frequency tables, graphs, and descriptive summaries were used to describe the study variables. Bivariate analysis was performed for each variable to select variables candidate's for multivariate analysis. Multiple logistic regression analysis was used to control the effect of confounding variables and to identify associated risk factors for prevalence of tuberculosis. Independent sample *t*-test was used to compare the means of continuous variables between TB cases and non-TB cases. A 95% CI and *p* value less than 0.05 in multivariate analysis were considered as statistically significant.

### 2.6. Data Quality Assurance

To ensure the quality of data different quality control activities were in place. Standardization of procedures and providing training for data collectors and periodic supervisions were conducted. All laboratory tests were done by strictly following the standard operation procedures (SOPs) and manufacturer instructions.

Reagents were checked for their expiry date before any analysis is started and H37Rv strain was used to check the performance of 5% MGIT^960^ tube batches for supporting the growth of* M. tuberculosis* and to check the quality of ZN staining reagents. Blood agar was prepared by strict aseptic technique and 5% of these prepared media were checked for sterility by putting them in the incubator at 37°C for 48 hours. Standard gram-positive and gram-negative bacteria were used to check the performance of sample blood agar for supporting the growth of bacteria.

### 2.7. Ethical Considerations

Ethical clearance was obtained from Institutional Review Board (IRB), College of Health Sciences, JU, Jimma. Participants were informed about the purpose and procedures of the study and consent was obtained from each study participant. All information obtained from participants during the study was kept confidential.

## 3. Results

### 3.1. Characteristics of Study Participants

Out of 129 participants 99 (76.7%) were males. The mean age in years of the participants was 22.5 ± 2.6 standard deviation. More than three-fourths 99 (76.7%) of the participants were male. Most 107 (82.9%) of participants were single. Twenty-seven (20.9%) of study participants were smokers. Forty-nine (38.0%) of study participants were alcohol drinkers. Furthermore, 52 (40.3%) of the participants were khat chewers. Almost one-fourth 36 (27.9%) of the participants were third year students. One hundred seven (82.9%) of study participants shared the dormitory with six or more students. Forty (31.0%) of the participants had contact history with TB patients. Majority 87 (67.4%) of participants had a BMI value of above or equal to 18.5 kg/m^2^.

### 3.2. Magnitude of Tuberculosis

Of 129 TB suspected participants, 41 [31.8% (95% CI: 22.7–40.9%)] were confirmed to have tuberculosis. Considering the total number of undergraduate students (19,598) attending Jimma University during 2014/2015 academic year, the overall prevalence estimate of TB was 209.2 per 100,000-student population.

From the total 41 TB cases identified 31 (75.6%) and 10 (24.4%) were pulmonary tuberculosis and extrapulmonary tuberculosis cases, respectively. Of 31 patients with PTB, 28 (90.3%) were bacteriologically confirmed cases (smear and/or culture positive) and 3 (9.7%) were diagnosed by chest X-ray. Of the total 10 EPTB cases, 7 (70.0%) were diagnosed by FNA cytology and 3 (30.0%) of them were diagnosed by culture.

### 3.3. Morbidity Related Conditions

The leading clinical symptoms reported were cough 119 (92.24%), weight loss 67 (51.93%), chest pain 66 (51.16%), fatigue 63 (48.83%), loss of appetite 61 (47.28%), shortness of breath 56 (43.41%), night sweat 46 (35.66%), and bloody sputum 12 (9.30%). The mean duration of cough was 4.20 weeks (95% CI: 3.75–4.96).

### 3.4. Factors Associated with Tuberculosis

The univariate analysis of the association between sociodemographic and behavioral factors and tuberculosis infection among students was summarized in Table 1. The mean age of TB cases (22.71 ± 2.09 year) was not different from those suspects (22.39 ± 2.90 year) (*t*-test = 0.523). It is observed that tuberculosis is significantly higher on students who had a habit of cigarette smoking, khat chewing, and alcoholic consumption (Table 1). The mean number of students per dorm was 6.95 ± 1.96 and the average space occupied in dorm per student was about 2.5 sq meters. On top of these, neither number of students per dorm nor availability of window was associated with active tuberculosis disease (Table 1).

The mean overall body mass index (BMI) of the study participants was 19.8 kg/m^2^. Likewise, the mean BMI among TB cases (18.61 ± 2.09 kg/m^2^) was significantly lower than non-TB participants (20.30 ± 2.37 kg/m^2^) [*t*-test, *p* < 0.001, OR = 4.64 (*p* = 0.001, 95% CI: 2.09–10.32)] (Table 1). Close contact with TB patient was also significantly associated with TB (*p* value = 0.011, OR (95% CI) = 2.76 (1.26–6.04)) (Table 1).

### 3.5. Multivariate Logistic Regression Analysis of TB Predictors

In this study, cigarette smoking, khat chewing, and low body mass index were significantly associated with active TB (Table 2).

## 4. Discussion

Ethiopia has achieved the MDG target to halt and reverse TB incidence and now focus on achieving the targets set in the new End TB Strategy. Though significant achievements were recorded to control TB in Ethiopia, the numbers of tuberculosis cases remain high in the country [2]. In our study, the result showed that the magnitude of all form of TB among JU undergraduate students was 209.2 per 100000-student population. In comparison with previous retrospective studies published in Ethiopia, our finding is lower than overall calculated prevalence rate of 354.66 from Gondar University [14], 1098.1 reported from Addis Ababa Science and Technology University, and 521.6 reported from Addis Ababa University by Gebrehiwot et al., 2016 [15]. This difference in prevalence might be associated with the retrospective study design methodology of these two studies, the time interval between this study and the fore-mentioned retrospective study and geographic difference. On the other hand the overall prevalence of TB observed in this study is consistent with 2015 WHO Ethiopian population TB prevalence report [2].

This study has also revealed that some factors including BMI, smoking, and khat chewing are independently associated with TB among Jimma University students.

In Ethiopia, khat chewing is becoming a great problem among young adults [17, 18]. University students are usually perusing their education far from their home and parents. They assume every decision of their life by themselves without parental influence. As a result university students are usually tempted to try new experiences which otherwise were difficult to try when they are living with their parents. Most of the students start khat chewing, smoking, and alcohol consumption during their stay in the universities [17]. It should be noted here that 40% and 20% of students participated in this study were khat chewers and smokers, respectively.

Khat chewing is usually the core of the problem. In most of the cases it drives the chewer to smoke and drink alcohol too. Focused analysis done in this study showed that students who chew khat are also four times more likely to smoke cigarette and drink alcohol. As a consequence, most of them lose their appetite which in turn leads to loss of weight and immune suppression. Some of the chemical contents of khat were also described before to cause loss of appetite [19]. This may create an opportunity for TB bacilli to initiate infection or reactivation.

The other observed determinant was BMI. Low body mass index (<18.5 kg/m^2^) was positively associated with active TB. Students with body mass index less than 18.5 kg/m^2^ have an increased risk of TB than those students with BMI ≥ 18.5 kg/m^2^. This finding is supported by previous study from Ethiopia [20]. A systematic review conducted by Lönnroth et al. [21] also revealed the presence of a strong and consistent log-linear relationship between TB incidence and BMI.

It is true that weight loss due to nutrition deficiency results in a range of changes to macro- and micronutrient status. This change adversely affects precisely those immunological mechanisms that are crucial for the successful control of mycobacterium. For example, it is described that dietary deficiency of micronutrients like zinc, vitamin D, vitamin A, vitamin C, and iron can cause profound impairment of immunity that are critical to “fight” tuberculosis [22]. Moreover, one of the ingredients of khat cathinone is shown to have immunomodulatory effect which might further increase the vulnerability of khat chewers for TB [19]. Previous studies done in Ethiopia also described the significant relation between TB with smoking and khat chewing [18, 23].

One of the limitations of current study is inability to conduct dormitory to dormitory active TB case finding in the university due to logistic and time constraints. As a result we might have missed some TB cases who could not present themselves to student clinics. Hence, the observed overall prevalence of TB among Jimma University student might be underestimated. In addition, the small sample size of this study might have underestimated or overestimated the prediction of risk factors for TB.

In conclusion tuberculosis is a significant problem in Jimma University undergraduate students. Cigarette smoking, khat chewing, and lower BMI were independent predictors for active TB. Thus, enough emphasis should be given to control TB in universities. Further study with active case finding approach to further explore the problem is recommended.

## Figures and Tables

**Table 1:** Univariate analysis of the association between TB cases and associated factors.

Variables	Tuberculosis status		OR (95% CI)	*p* value
	Positive number (%)	Negative number (%)		
Sex				
Male	35 (35.4)	64 (64.6)	2.19 (0.82, 5.86)	0.12
Female	6 (20.0)	24 (80.0)	1.00	
Age category				
>25	5 (31.2)	11 (68.8)	2.84 (0.64, 12.65)	0.17
20–25	32 (38.1)	52 (61.9)	3.85 (1.23, 12.07)	0.01^*∗*^
<20	4 (13.8)	25 (86.2)	1.00	
Year of study				
>4th year	4 (28.6)	10 (71.4)	1.36 (0.29, 6.28)	0.69
4th year	14 (41.2)	20 (58.8)	2.38 (0.71, 7.97)	0.16
3rd year	15 (41.7)	21 (58.3)	2.43 (0.73–8.04)	0.15
2nd year	3 (13.0)	20 (87.0)	0.51 (0.11, 2.45)	0.40
1st year	5 (22.7)	17 (77.3)	1.00	
Number of students per dorm				
6 or more	37 (34.6)	70 (65.4)	2.34 (0.75, 7.55)	0.14
4-5 students	4 (18.2)	18 (81.8)	1	1
Availability of window				
Yes	32 (29.9)	75 (70.1)	0.62 (0.24, 1.59)	0.32
No	9 (40.9)	13 (59.1)	1	
Cigarette smoking				
Yes	19 (70.4)	8 (29.6)	8.64 (3.34–22.36)	0.001^*∗*^
No	22 (21.6)	80 (78.4)	1.00	
Alcohol consumption				
Yes	22 (44.9)	27 (55.1)	2.62 [1.22–5.61]	0.013^*∗*^
No	19 (23.8)	61 (76.2)	1.00	
Khat chewing				
Yes	26 (50.0)	26 (50.0)	4.13 (1.89–9.05)	0.001^*∗*^
No	15 (19.5)	62 (80.5)	1.00	
Previous hospitalization				
Yes	2 (18.2)	9 (81.8)	0.45 (0.09, 2.18)	0.322
No	39 (33.1)	79 (66.9)	1.00	
BMI (kg/m^2^)				
<18.5	23 (54.8)	19 (45.2)	4.64 (2.09, 10.32)	0.001^*∗*^
≥18.5	18 (20.7)	69 (79.3)	1.00	
TB contact history				
Yes	19 (47.5)	21 (52.5)	2.76 (1.26, 6.04)	0.011
No	22 (24.7)	67 (75.3)	1.00	

1.00 = referent category; ^*∗*^statistically significant.

**Table 2:** Univariate and multivariate analysis of some factors associated with tuberculosis.

Variables	COR (95% CI)	*p* value	AOR (95% CI)	*p* value
Male gender	2.19 (0.82–5.86)	0.119	1.39 (0.39–4.92)	0.607
Age	1.74 (0.90–3.36)	0.099	2.14 (0.91–5.03)	0.082
History of contact (yes)	2.76 (1.26–6.04)	0.011	2.26 (0.84, 6.08)	0.106
Number of students per dorm (≥6)	2.38 (0.75–7.54)	0.141	2.97 (0.76–11.58)	0.117
Cigarette smoking (yes)	8.64 (3.34–22.36)	0.001	4.30 (1.35–13.69)	0.013^*∗∗*^
Alcohol consumption (yes)	2.62 (1.22–5.61)	0.013	0.94 (0.33–2.72)	0.915
Khat chewing (yes)	4.13 (1.89–9.05)	0.001	2.86 (1.09–7.48)	0.032^*∗∗*^
BMI (kg/m^2^)	0.69 (0.56–0.85)	0.001	0.73 (0.58–0.94)	0.015^*∗∗*^

COR = crude odds ratio, AOR = adjusted odds ratio, and ^*∗∗*^statistically significant.
